# Micro-tip Cantilever as Low Frequency Microphone

**DOI:** 10.1038/s41598-018-31062-9

**Published:** 2018-08-23

**Authors:** Sumit Dass, Rajan Jha

**Affiliations:** Nanophotonics and Plasmonics Laboratory, School of Basic Sciences, Indian Institute of Technology Bhubaneswar, Khurda, 752050 India

## Abstract

We propose a very compact diaphragm free optical microphone consisting a tapered micro-tip in cantilever configuration for detection of low frequency acoustic signals. The change in the light coupling between the micro-tip and the source fiber caused by the acoustic pressure is utilized to detect the external acoustic signal. The sensitivity and working range of the sensor depend on three key factors, the length of the micro-tip cantilever, the distance between the micro-tip and SMF, and the offset between the micro-tip central axis and SMF central axis. Hence, by changing any of these parameters, the performance of the sensor can be easily tuned. Experimental results show that for a cantilever length of 15 mm, the probe has a maximum acoustic sensitivity of 10.63 mV/Pa or −159.5 dB re 1 V/μPa, noise-limited minimum detectable pressure of 19.1 mPa/√Hz and the linear frequency range is 0–400 Hz. The SMF only structure along with photodetector-based interrogation makes this acoustic sensor economical.

## Introduction

Low frequency acoustic signal detection is one of the major fields of interest owing to its application in the field of differential acoustic resonance spectrometry (DARS), cervical auscultation and low frequency vibro-acoustic analysis^1–3^. The results of these applications are highly dependent on the number of sensing element per unit area, and the signal to noise ratio of the sensor. In all of these applications, the commercialized microphones based on piezoelectric technology has been used. The miniaturization of piezoelectric microphones comes at the cost of higher noise level and very complex fabrication. Additionally, piezoelectric sensors have an inability to be directly employed for underwater applications and in areas where high electromagnetic fields are present. In the last decade, a range of optical fiber based acoustic sensors have been proposed which exhibit equal or even better performance as compared to traditional piezoelectric microphones in terms of sensitivity, bandwidth and noise-limited minimum detectable pressure^4–6^. Furthermore, optical fiber based sensors have inherent advantages of multiplexing, remote sensing, immunity to electromagnetic signals and they can be used as hydrophones for underwater applications^7^.

On a broader basis, most of the optical fiber microphones (OFMs) use diaphragms as a part of sensing element; where a thin reflecting diaphragm is used to amplify the effect of acoustic signal^8–10^. Very limited diaphragm free OFMs are reported including the work reported by Huang *et al*. where they have demonstrated a polymer micro-ring resonator based OFM for detection of ultrasound^11^. The fabrication of micro-ring resonator is done by complicated and costly nano-imprinting technique. Mach- Zehnder based interferometric structure including polarization-maintaining photonic-crystal-fiber (PM-PCF), have been demonstrated as hydrophone^12^. Cranch *et al*. have demonstrated hydrophone who’s sensor structure includes fiber laser and fiber Bragg grating (FBG)^13^. These sensing schemes require high end equipment to detect phase/wavelength and are susceptible to noise caused by temperature and pressure induced variations in phase/wavelength. Additionally, the complex optical design along with the requirement of nano-imprinting system, fiber laser, PM-PCF and FBG adds to the cost of the device fabrication.

In order to overcome the complexity involved with diaphragm fabrication and to avoid the requirement of costly phase/wavelength measuring devices, we propose a very compact diaphragm free acoustic sensor consisting an optical fiber micro-tip in cantilever configuration. The sensor system is quite easy to fabricate, cost effective and fairly tunable in terms of working range and sensitivity. Detailed theoretical and experimental analysis is done on the performance of our acoustic sensor for different cantilever lengths and the experimental results are in good agreement with theoretical analysis.

## Sensor Fabrication and Working Principle

**Figure 1:**
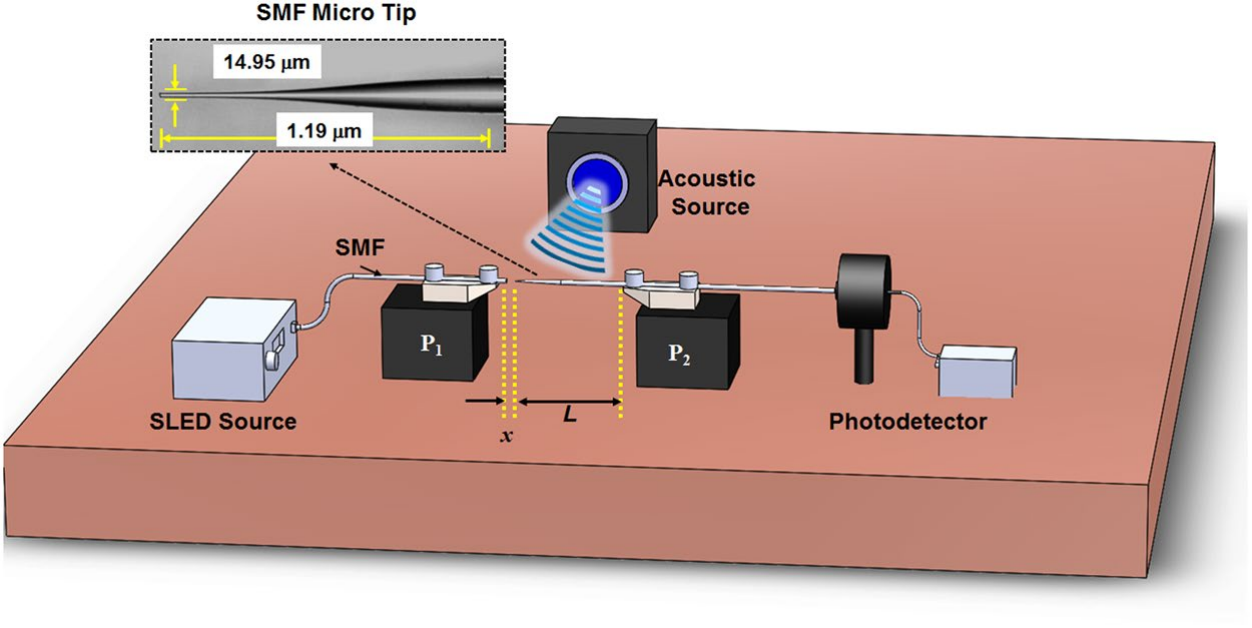
Schematic of micro-tip cantilever based optical fiber microphone; Inset shows the microscopic image of the SMF micro-tip.

The proposed Micro-tip Cantilever Microphone (MCM) setup consist a micro-tip in cantilever configuration, as shown in Fig. 1. To fabricate the micro-tip, firstly a simple single mode fiber (SMF) taper is fabricated using well established flame and brush technique^14^. The SMF taper is then carefully cleaved at the waist position where the fiber diameter is smallest. By doing so, we create two micro-tips. The inset of Fig. 1 shows the micrograph of one such micro-tip with a tip diameter of 14.95 μm and length of micro-tip is 1.19 mm. The tapered micro-tip is tightly clamped on a V-groove attached to a computer-controlled stage *P*_1_ in such a way that it creates a cantilever of length ~15 mm, as shown in the Fig. 1. The other end of micro-tip fiber is connected to the photodetector system. A standard SMF with vertically cleaved end is placed in front of the tapered micro-tip on a V-groove attached to another computer-controlled stage *P*_2_ and tightly clamed. The other end of vertically cleaved SMF is connected to a Superluminescent Light Emitting Diode (SLED) source. The broadband light generated by the SLED source comes out of the 8 μm core of the SMF and gets coupled to the tapered micro-tip placed at 10 μm distance.

**Figure 2:**
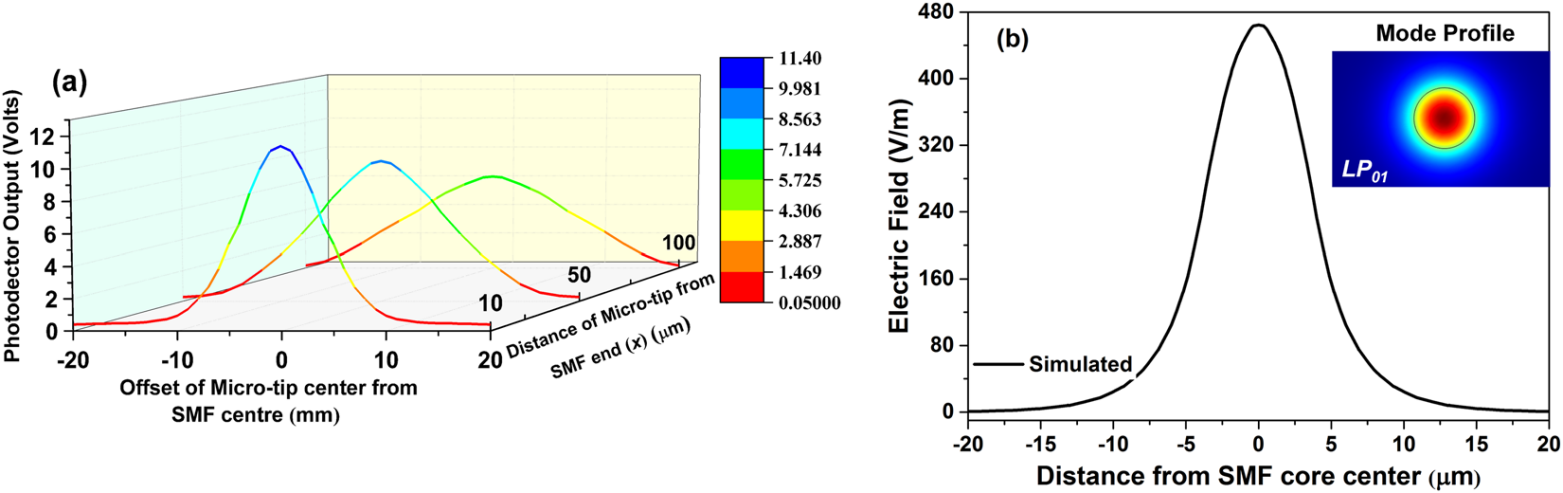
(**a**) The variation of micro-tip coupled light at different offset positions for different distance between micro-tip and SMF end; (**b**) simulated output electric field at SMF end with distance from the center of the SMF core; and inset of (**b**) shows the simulated mode profile of the standard SMF.

While keeping the output power of SLED constant, the amount of light which gets coupled to the guided mode of micro-tip depends on two parameters; the distance between micro-tip and cleaved end of SMF (***x***) and the offset between the central axial of micro-tip and cleaved end of the SMF. Figure 2(a) shows the experimental variation of the photodetector output when the micro-tip was placed at different ***x*** and at different offset positions. As it can be seen, the photodetector output for distinct values of ***x*** follow Gaussian distribution curve. This distribution is similar to that of the fundamental mode (*LP*_01_) profile of standard SMF shown in the inset of Fig. 2(b), and the variation of electric field along the core axis is plotted in Fig. 2(b) by black line. The results shown in Fig. 2(b) are generated using Finite Element Method.

**Figure 3:**
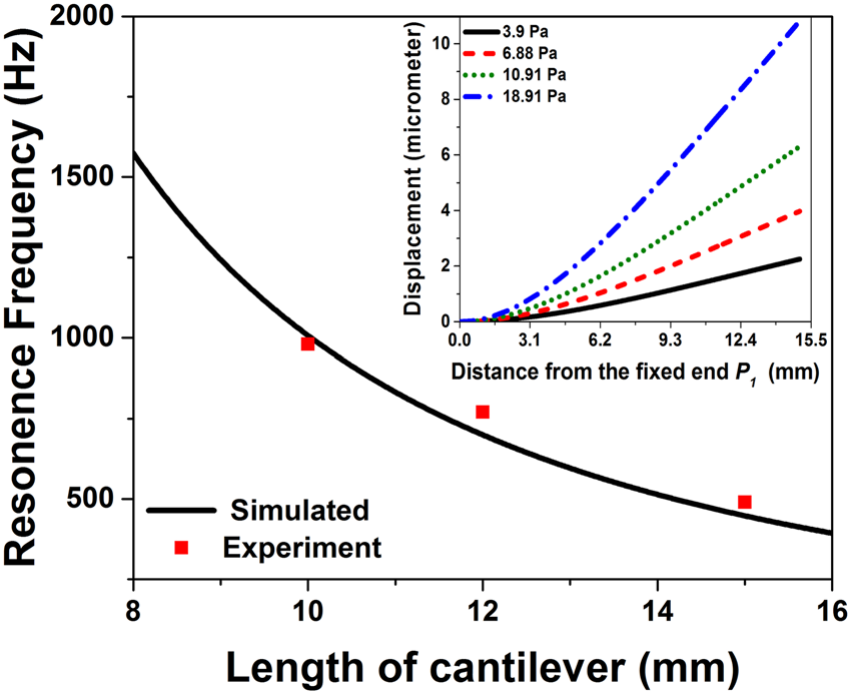
Variation of resonance frequency with cantilever length; Inset shows the displacement of micro-tip cantilever under different applied acoustic pressure.

The experimental results are well in agreement with the simulated results and clearly indicate that the power coupled to the micro-tip is very much sensitive to the displacement of micro-tip from the SMF central axis. When an external acoustic field is applied to the micro-tip cantilever setup, the airborne acoustic pressure waves cause a minuscule displacement of micro-tip which leads to a change in the photodetector output. Hence, given a proper cantilever length the tapered micro-tip can be used to sense the applied acoustic signal. As the gradient of the coupled light field is maximum at offset positions of 4 μm, 6 μm and 7 μm, respectively for ***x*** = 10 μm, 50 μm and 100 μm, the acoustic sensitivity can also be expected to be maximum at these positions. To analyse the effect of acoustic pressure on the tapered micro-tip cantilever, finite element method is used to model the structure and its performance is studied under variable acoustic pressure and the results are shown in the inset of Fig. 3. As it can be seen that an increase in applied acoustic pressure causes more deflection of fiber micro-tip. The maximum deflection (*Δl*_*max*_) of a cylindrical cantilever for an applied pressure (*P*) is defined as^15^,

1 $${\rm{\Delta }}{{l}}_{{\rm{\max }}}=\frac{{P}{{L}}^{4}}{2\pi {Ed}}$$

where, *L* is the length of cantilever, *E* is the Young’s modulus of the cantilever material and *d* is diameter of the cantilever. It is clear from Eq. (1) that to achieve higher values of end deflection, the length of cantilever needs to be increased. However, by doing so the first order natural frequency (*f*_*nf*_), defined below, will decrease^15^.

2 $${{f}}_{{nf}}=\frac{{(1.875)}^{2}}{8\pi }\sqrt{\frac{{E}{{d}}^{2}}{{\rho }{{L}}^{4}}}$$

where, *ρ* = 2230 kg/m^3^ is the material density of the silica cantilever and the Young’s modulus (*E*) for silica is 73.1 GPa. With these values, the calculated first order natural frequency with varying cantilever length is plotted and shown in Fig. 3.

## Results and Discussion

**Figure 4:**
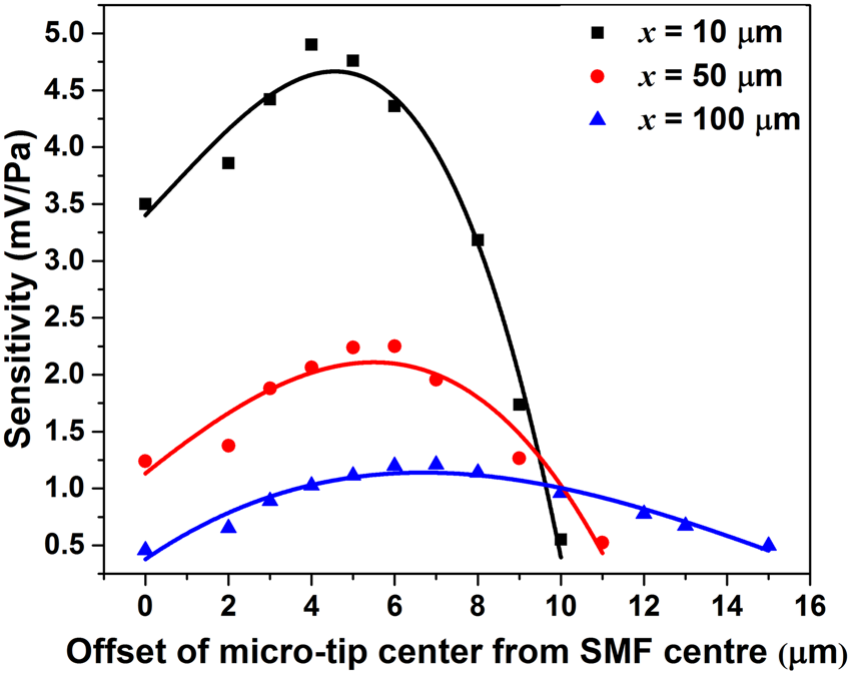
The experimental sensitivity variation with different offset values at different distances of micro-tip from SMF end (***x***).

To examine the acoustic performance of such a micro-tip based cantilever structure, an acoustic source is placed in the horizontal plane at a distance from the MCM setup having a cantilever length of *L*, as shown in Fig. 1. For referencing, a commercial microphone (B&K) is placed just beside the MCM setup. The proposed MCM and reference microphone setups are placed equidistant to the acoustic source. Acoustic waves generated from the acoustic source deflect the micro-tip of the fiber cantilever structure. This deflection changes the amount of optical power coupled to the micro-tip which is recorded using a photodetector (Thor Labs). In order to find the most optimal working point, we tested the performance of the sensor at different offset positions. The resultant sensitivity variations of the sensor with 12 mm cantilever length for a 250 Hz acoustic signal are shown in Fig. 4. As it can be seen, the sensor sensitivity increases at first with an increase in offset, then it reaches the maxima corresponding to the largest gradient of the coupling light intensity. The maximum sensitivity points are at offset positions of 4 μm, 6 μm and 7 μm, respectively for ***x*** = 10 μm, 50 μm and 100 μm. Further increasing the offset decreases the sensitivity due to the lower gradient of the coupling light intensity.

**Figure 5:**
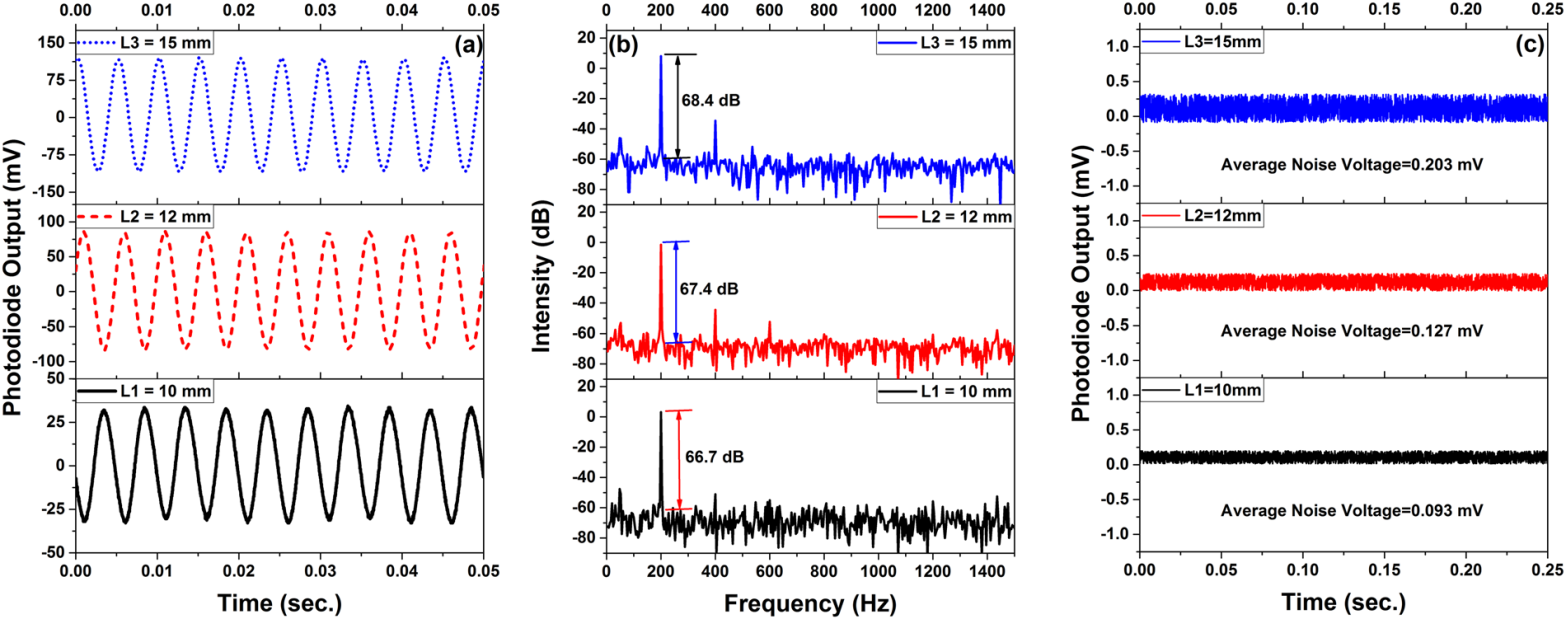
(**a**) Time domain and (**b**) frequency domain response for an external acoustic signal of 200 Hz, and (**c**) output voltage of the photodetector in the absence of acoustic signal, for the three MCMs with different cantilever lengths.

At these optimal positions, the MCM setups with different cantilever length 10 mm (*L*_1_), 12 mm (*L*_2_) and 15 mm (*L*_3_) are tested with acoustic signals of different frequency and intensity. Figure 5(a,b) show the time domain and frequency domain outputs of the photodetector for the MCMs with different cantilever lengths for an acoustic signal of 200 Hz. Further, Fig. 5(c) represents the average noise associated with MCMs of different cantilever lengths.

**Figure 6:**
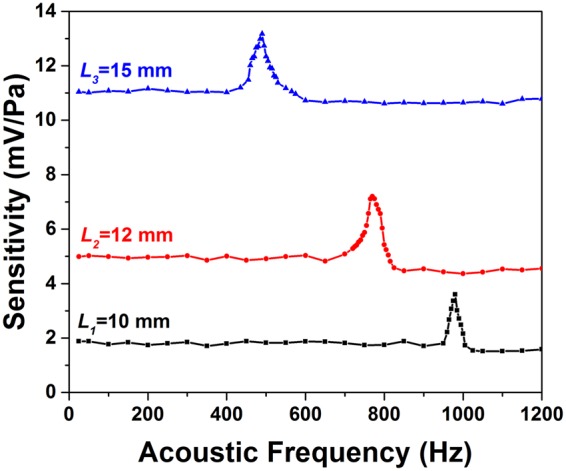
The variation of acoustic sensitivity with frequency for the three sensors with different cantilever length.

As it can be observed from Fig. 5(b), for an applied acoustic signal of 200 Hz, the signal to noise ratio (SNR) for MCMs with *L*_3_, *L*_2_ and *L*_1_ are 68.4 dB, 67.4 dB and 66.7 dB respectively. The acoustic sensitivity of MCMs are calculated with the help of signal recorded by the reference microphone. The variation of sensitivity with acoustic frequency for all the three MCM setups are shown in Fig. 6.

As one can observe, for all the cantilever lengths, the sensitivity is nearly constant for lower acoustic frequencies and then it starts increasing rapidly to reach a maximum value, following which the sensitivity again starts decreasing. The point of maximum sensitivity corresponds to the first order natural frequency (*f*_*nf*_) of the cantilever structure. The experimental values of the first order natural frequency (*f*_*nf*_) are 490 Hz, 770 Hz and 980 Hz, respectively for cantilever lengths of *L*_3_, *L*_2_ and *L*_1_. These experimental values are in good agreement with the values calculated by Eq. (2), as shown in Fig. 3. The small variation between the theoretical and experimental values of *f*_*nf*_ can be attributed to the fact that unlike the experimental structure, for theoretical calculation we have considered a constant diameter of 125 μm for the entire cylindrical cantilever. Experimental results suggest that the linear working frequency range for MCM with cantilever length *L*_3_ to be 0–400 Hz, 0–700 Hz for the cantilever length *L*_2_, and 0–950 Hz for the cantilever length *L*_1_. Therefore, the working range of the sensor can be tailored by appropriately choosing the cantilever length as per the application which is a unique advantage of the proposed device.

**Figure 7:**
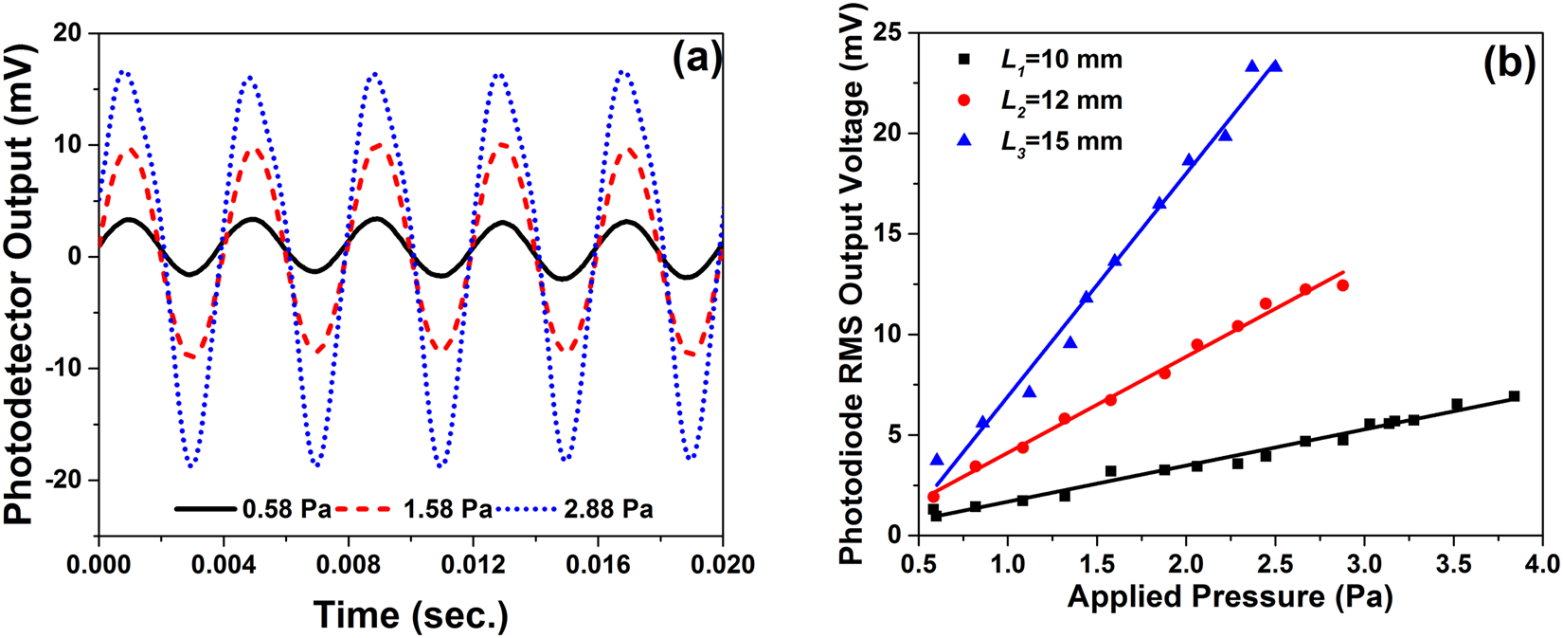
(**a**) Time domain response recorded for MCM with cantilever length 12 mm with different acoustic pressures at 250 Hz; and (**b**) Variation of photodiode RMS voltage (*V*_*rms*_) with different applied acoustic pressure at 250 Hz.

To study the response of MCM for different level of acoustic pressure, acoustic signals of fixed frequency (250 Hz) and varying intensity (0.5 Pa to 5 Pa) is generated. Figure 7(a) shows some of the photodetector output recorded during such an experiment for MCM with cantilever length *L*_2_ at ***x*** = 10 μm with an offset of 4 μm. With increasing acoustic pressure, the micro-tip deflection also increases (as shown in the inset of Fig. 3), hence the RMS voltage (*V*_*rms*_) of the output signal recorded by photodetector also increases. Similar experiment is performed for two other cantilever lengths, *L*_1_ and *L*_3_. The photodiode RMS output voltage (*V*_*rms*_) corresponding to different acoustic pressure is plotted in Fig. 7(b), for all the three cantilever lengths. The variation in *V*_*rms*_ is linear for starting values of applied pressure and the linear range of the three sensor setups are 0–2.5 Pa, 0–2.88 Pa and 0–3.83 Pa respectively for sensors with cantilever lengths of *L*_3_, *L*_2_ and *L*_1_. The slope the data points of the linear range give the sensitivities of the sensors which are 10.63 mV/Pa, 4.61 mV/Pa and 1.88 mV/Pa or −159.5 dB re 1 V/μPa, −166.7 dB re 1 V/μPa and −174.5 dB re 1 V/μPa. The experimental results clearly indicate that with decreasing cantilever length, the acoustic sensitivity increases whereas the first order natural frequency of the related structure decreases. This decrease in natural frequency limits the linear working range of the sensor. In order to calculate the noise-limited minimum detectable pressure (MDP), we recorded the noise signal in absence of applied acoustic pressure, as shown in Fig. 5(c). With an average noise signal for cantilever lengths *L*_3_, *L*_2_ and *L*_1_ as 0.203 mV, 0.127 mV and 0.093 mV, the noise-limited MDP can be calculated as 19.1 mPa/√Hz, 30.5 mPa/√Hz and 49.5 mPa/√Hz, respectively. The acoustic sensitivity and MDP of the present micro-tip cantilever based system is better than the acoustic sensors reported by Wang *et al*.^16^, Luo *et al*.^17^, Chen *et al*.^18^, Minasamudram *et al*.^19^ and Kim *et al*.^20^.

## Conclusion

In conclusion, we have successfully designed, developed and demonstrated the operation of a simple, low-cost, micro-tip cantilever system and its use in low frequency acoustic signal detection. The experimental results which are well in agreement with theoretical results, show that the performance of the proposed optical microphone can be easily tuned by changing the length of cantilever, the distance between the micro-tip and SMF, and the offset between the micro-tip central axis and SMF central axis. With increasing cantilever length, the sensitivity increases and the signal to noise ratio decreases whereas the change in the distance between the micro-tip and SMF have an inverse effect on sensitivity. For every cantilever setup there is an optimal offset position of micro-tip which gives the maximum sensitivity. Optical microphone with cantilever length of 15 mm shows a sensitivity of 10.63 mV/Pa or −159.5 dB re 1 V/μPa at 250 Hz and a noise-limited minimum detectable pressure of 19.1 mPa/√Hz. The proposed optical microphone will be of high utility for acoustic level monitoring in workplaces like foundries, saw /textile/crushing mills and aircraft maintenance workshops.
